# A vigilance decrement comes along with an executive control decrement: Testing the resource-control theory

**DOI:** 10.3758/s13423-022-02089-x

**Published:** 2022-04-27

**Authors:** Fernando G. Luna, Miriam Tortajada, Elisa Martín-Arévalo, Fabiano Botta, Juan Lupiáñez

**Affiliations:** 1 Instituto de Investigaciones Psicológicas (IIPsi, CONICET-UNC), Facultad de Psicología, Universidad Nacional de Córdoba, Boulevard de la Reforma esquina Enfermera Gordillo, CP 5000 Córdoba, Argentina; 2 Department of Experimental Psychology, and Mind, Brain, and Behavior Research Center (CIMCYC), University of Granada, Campus de Cartuja S/N, CP 18011 Granada, Spain; 3 Present Address: Department of Basic Psychology and Methodology in Faculty of Psychology, University of Murcia, Campus Universitario de Espinardo, CP 30100 Murcia, Spain

**Keywords:** Vigilance decrement, Resource-control, Executive control, Executive vigilance, Arousal vigilance

## Abstract

A decrease in vigilance over time is often observed when performing prolonged tasks, a phenomenon known as “vigilance decrement.” The present study aimed at testing some of the critical predictions of the resource-control theory about the vigilance decrement. Specifically, the theory predicts that the vigilance decrement is mainly due to a drop in executive control, which fails to keep attentional resources on the external task, thus devoting a larger number of resources to mind-wandering across time-on-task. Datasets gathered from a large sample size (*N* = 617) who completed the Attentional Networks Test for Interactions and Vigilance—executive and arousal components in Luna, Roca, Martín-Arévalo, and Lupiáñez (2021b, *Behavior Research Methods*, *53*[3], 1124–1147) were reanalyzed to test whether executive control decreases across time in a vigilance task and whether the vigilance decrement comes along with the decrement in executive control. Vigilance was examined as two dissociated components: executive vigilance, as the ability to detect infrequent critical signals, and arousal vigilance, as the maintenance of a fast reaction to stimuli. The executive control decrement was evidenced by a linear increase in the interference effect for mean reaction time, errors, and the inverse efficiency score. Critically, interindividual differences showed that the decrease in the executive—but not in the arousal—component of vigilance was modulated by the change in executive control across time-on-task, thus supporting the predictions of the resource-control theory. Nevertheless, given the small effect sizes observed in our large sample size, the present outcomes suggest further consideration of the role of executive control in resource-control theory.

**Supplementary Information:**

The online version contains supplementary material available at 10.3758/s13423-022-02089-x.

It is well known that sustained attention declines in prolonged tasks—a phenomenon known as “vigilance decrement”. The vigilance decrement is usually observed as a progressive slowness in responses as well as a drop in the correct detection on infrequent critical signals (i.e., hits) with time-on-task (Adams, 1987; Hancock, 2017; Mackworth, 1948). However, although the vigilance decrement has been extensively studied for decades (Esterman & Rothlein, 2019; Fortenbaugh et al., 2017; Thomson et al., 2015), there is still an open debate concerning the theoretical explanations underlying the mechanisms leading to a progressive loss of vigilance across time-on-task.

Nowadays, the predominant account regarding the vigilance decrement is the “resources depletion” hypothesis (Hancock, 2017; Warm et al., 2008). Based on the assumptions that attention works as a limited pool of resources that cannot be continuously reloaded and that vigilance tasks are difficult to perform and demand hard mental work, the resources depletion account predicts that as time-on-task progresses, attentional resources are progressively depleted, thus leading to a loss of vigilance (Grier et al., 2003; Warm et al., 2008). An alternative account to this model is related to mind-wandering (Smallwood, 2010; Smallwood & Schooler, 2006). From this alternative view, attentional resources are not just depleted but rather shared between the external task being performed and internal task-irrelevant thoughts unnecessary to complete the task at hand (Murray et al., 2020). The mind-wandering account anticipates that as time-on-task progresses, there is an increasing difficulty to keep attentional resources on the task being performed so that attentional resources are redirected to task-irrelevant thoughts, thus leading to a progressive disengagement with the task at hand and, consequently, to a loss on vigilance (Konishi et al., 2017; Stawarczyk et al., 2011; Thomson et al., 2014).

In the last years, the “resource-control” theory has been proposed as a new alternative model concerning the vigilance decrement by, importantly, considering predictions from both the resources depletion and the mind-wandering account (Thomson et al., 2015). According to the resource-control theory, mind-wandering is our default state, there being a continuous bias towards this state. Nevertheless, mind-wandering consumes attentional resources that cannot be devoted to the vigilance task performed at hand (Smallwood, 2010; Smallwood & Schooler, 2006). Therefore, executive control is necessary to keep the task’s objectives in mind and to prevent an increasing emergence of task-irrelevant thoughts. Importantly, the resource-control theory posits that attentional resources are fixed in each individual and do not change throughout the task. Instead, there is a drop in executive control across time-on-task, which consequently leads to misappropriation of attentional resources by mind-wandering processes. Thus, as fewer resources are devoted to the external task than to mind-wandering across time-on-task (Thomson et al., 2015), there is a progressive increase in performance costs that is reflected as a decrease in vigilance.

Although the resource-control theory might provide an appropriate framework to explain the vigilance decrement (Thomson et al., 2015), empirical evidence on this theoretical framework is still scarce. Most importantly, to the best of our knowledge, no study has directly examined whether there is a decrease of executive control along with the vigilance decrement with time-on-task. This concurrent decrement is a key distinguishing aspect of the resource-control theory, as no other previous theory of vigilance decrement hypothesized about executive control (see Table 2 in Thomson et al., 2015). Zholdassova et al. (2021) tried to test this theory, but they only analyzed whether there is a progressive drop in executive control across time-on-task and not in vigilance performance. By using the Attention Network Test (ANT; Fan et al., 2002), the authors examined phasic alertness, orienting, and executive control as a function of time-on-task. Importantly, they found no change in executive control with time-on-task, a piece of data against the resource-control theory. It is important to note, however, that the ANT is not a suitable task to observe vigilance decrement, as it is rather usual to observe improved performance with practice across time-on-task (Ishigami & Klein, 2010). Satterfield et al. (2019) investigated whether depleting executive control prior to completing a vigilance task had any impact on vigilance performance. The authors observed no difference in vigilance performance across time-on-task between executive-control-depleted and nondepleted participants, therefore interpreting these findings as evidence against the resource-control theory (Satterfield et al., 2019).

Nevertheless, to test the predictions derived from the resource-control theory, it seems necessary to examine whether the vigilance decrement is accompanied by a similar drop in executive control across time-on-task, a behavioral pattern that has not been directly studied by previous research (Satterfield et al., 2019; Zholdassova et al., 2021). Furthermore, note that whereas vigilance has been traditionally examined as an unitary concept indistinctly measured with reaction time (RT) tasks, such as the Psychomotor Vigilance Test (PVT; Lim & Dinges, 2008), or signal-detection tasks, such as the Sustained Attention to Response Task (SART; Robertson et al., 1997) and the Continuous Performance Test (CPT; Conners, 2000), these behavioral tasks might be indeed assessing dissociated components of vigilance (Oken et al., 2006; Sarter et al., 2001).

On the one hand, in the PVT the vigilance decrement is observed as a progressive increase in mean RT and RT variability to a single stimulus (i.e., a millisecond counter) that appears at random intervals of time. This task seems to assess vigilance as the maintenance of the arousal level of attention to keep a fast reaction to stimuli from the environment. Note that this arousal component of vigilance (AV) might be especially related to the arousal shifts during the sleep–wake cycle and therefore sensitive to the effects of sleep deprivation (Basner & Dinges, 2011; Lim & Dinges, 2008). On the other hand, in tasks such as the SART and the CPT the vigilance decrement is observed as a progressive drop in hits over infrequent critical signals (See et al., 1995; Thomson et al., 2016). This drop in hits over infrequent signals has been proposed as a proxy measure of the vigilance decrement in signal-detection tasks, as it captures the difficulty to sustain attention during prolonged periods for perceiving rare signals that are crucial to be detected (Hancock, 2017; Mackworth, 1948). Thus, such signal-detection tasks might be assessing an executive component of vigilance (EV), which is related to the maintenance of a task set across time-on-task to categorize stimuli from the environment as signal and noise and consequently, to execute a specific response to stimuli (Luna et al., 2018).

To assess executive control—as well as phasic alertness and orienting—along with EV and AV, Luna et al. (2018) recently developed the ANT for Interactions and Vigilance—executive and arousal components (ANTI-Vea). The ANTI-Vea is a modified version of the ANT that comprises three subtasks: (a) the ANTI, a flanker task combined with a warning signal and a spatial cueing paradigm suitable for measuring the classic attentional networks functions; (b) a signal-detection task similar to the SART (Robertson et al., 1997) suitable to assess the EV decrement; and (c) an adapted version of the PVT (Lim & Dinges, 2008) suitable to assess the AV decrement. Importantly, the ANTI-Vea has (a) proved to be a task suitable to assess the classic attentional networks along with the EV and AV decrement among different populations (Huertas et al., 2019; Luna, Barttfeld, et al., 2021a; Román-Caballero et al., 2021), (b) demonstrated acceptable reliability for measuring EV and AV both in the typical lab conditions as well as in an online session performed outside the lab  (Luna, Roca, et al., 2021b), and (c) shown to be a task suitable to examine dissociable mechanisms between EV and AV at the physiological (Sanchis et al., 2020) and neural (Luna, Román-Caballero, et al., 2020b) levels.

## The present study

The present research aimed at examining important predictions derived from the resource-control theory concerning the association between executive control and the vigilance decrement phenomenon. In particular, the main objective was to test whether the vigilance decrement is accompanied by a similar drop in executive control across time-on-task (Thomson et al., 2015). To this end, executive control and vigilance components were measured in a single session with the ANTI-Vea (Luna et al., 2018). This goal was achieved by reanalyzing raw data (Luna, Roca, et al., 2020a) gathered from a large sample size (*N* = 617) with the standard (i.e., performed in the typical lab conditions) and the online ANTI-Vea (Luna, Roca, et al., 2021b), which is publicly available in the Open Science Framework (http://osf.io/q85bu). Importantly, note that Luna, Roca, et al. (2021b) observed an interference effect as a measure of executive control as well as a linear decrement in EV and AV with both the standard and the online ANTI-Vea tasks.

Following the predictions derived from the resource-control theory (Thomson et al., 2015), if executive control decreases with time-on-task a progressive increase in the interference effect—measured with the flanker subtask embedded in the ANTI-Vea—across blocks should be observed. The interference effect is a valid index for measuring executive control performance, as it captures the ability to organize and execute plans for actions to achieve our goals by measuring the selection of the target from distractors within a perceptual grouping of similar stimuli (e.g., a string of five arrows, as in the flanker subtask in the ANTI-Vea; Awh et al., 2012; Egner, 2008). Usually, given the incompatibility of distractors, the interference effect is observed as larger mean RT and percentage of errors in incongruent than in congruent trials. In the present study, cognitive interference was measured with mean RT, percentage of errors, and the inverse efficiency score (IE)—a summary measure of both mean RT and accuracy (see the Method; see also Bruyer & Brysbaert, 2011; Vandierendonck, 2017)—as dependent variables.

First, to test the relationship between executive control and vigilance decrements hypothesized by the resource-control theory, we performed bi-variate correlations between linear slopes across blocks of executive control and vigilance components. According to the resource-control theory, a negative correlation between executive control and hits of EV decrements and positive correlations between executive control and AV decrements (mean and *SD* of RT, and lapses) would be expected. Although some significant Pearson’s correlations were observed in this vein, which were also observed as positive evidence in favor of the existence of a correlation by Bayesian analyses, it is important to note that the correlations were relatively small. Moreover, IE slope showed a very low reliability, which was problematic for performing correlational analyses. Consequently, analyses of variance on the vigilance decrement across time-on-task were performed to examine the interaction between executive control and vigilance decrements. Note that, however, the correlational analyses are presented in Supplementary Material.

To test whether the vigilance decrement is modulated by the decrement in executive control, the slope of the linear decrement in executive control (i.e., the increase in interference) for each participant was introduced as a covariate in the analysis of vigilance performance across blocks. This way it was possible to examine whether the change in executive control across time-on-task interacts with the vigilance decrement. Then, to determine the way executive control modulated the vigilance decrement, the sample was divided into tertiles according to the size of the executive control slope in the IE score to compare the vigilance decrement in the two extreme groups of participants—that is, those with executive control functioning being decreased (i.e., with a positive slope in the IE score) or rather with no decrement (i.e., with a negative slope in the IE score) across time-on-task. According to the resource-control theory (Thomson et al., 2015), it would be expected that those participants with large executive control decrement across time-on-task would show a larger vigilance decrement, whereas participants with no change in executive control across time-on-task should not show vigilance decrement, as executive control would help to constantly maintain attentional resources on the external task. Thus, this analysis allowed us to specifically test a critical prediction of the resource-control theory, in particular, whether or not executive control loss across time-on-task is associated with the vigilance decrement. Altogether, the present study expects to provide novel, relevant, and critical evidence concerning the mechanisms underlying the vigilance decrement and more specifically the predictions derived from the resource-control theory (Thomson et al., 2015).

## Method

### Participants

The present study analyzed data gathered from 617 participants (445 women; age: *M* = 22.78; *SD* = 5.24) reported in Luna, Roca, et al. (2021b) and available online (http://osf.io/q85bu), who performed either the standard ANTI-Vea in the typical lab conditions (*n* = 314) or the online ANTI-Vea through a publicly available website (https://www.ugr.es/~neurocog/ANTI/) somewhere outside the lab (*n* = 303). Details of the participant’s groups who performed either the standard or the online ANTI-Vea (i.e., sex distribution, mean age, and location) can be reviewed in Table 1 of Luna, Roca, et al. (2021b). Importantly, given that no significant modulation of task version (standard vs. online) was observed over the executive control score, the linear decrement on hits for EV, and the linear decrement on the AV measures, in the present study we decided to reanalyze data from all participants as a single group, with no distinctions between task versions. The study was conducted according to the ethical standards of the 1964 Declaration of Helsinki (last update Seoul, 2008) and was part of a larger research project positively evaluated by the University of Granada Ethical Committee (536/CEIH/2018).

### Apparatus and stimuli

The standard ANTI-Vea was designed and run in E-Prime (Version 2.0 Professional, Psychology Software Tools, Pittsburgh, PA), and the online ANTI-Vea was designed and run using JavaScript ES5, HTML5, CSS3, and Angular JS. Both task versions used the same stimuli: a black fixation point (~7 pixels [px]), a black asterisk as visual cue (~13 px), a set of five arrows (~50 px wide and ~23 px high each of them) as target and distractors aligned as a horizontal vector, a tone as warning signal (2000 Hz), and a red milliseconds counter (~110 px height each number). Stimuli and instructions were presented over a grey background in a screen resolution of 1,024 px wide × 768 px high. In the standard ANTI-Vea, participants sat ~50 cm away from the screen and responses were collected on a standard QWERTY keyboard.

### Procedure and design

**Fig. 1:**
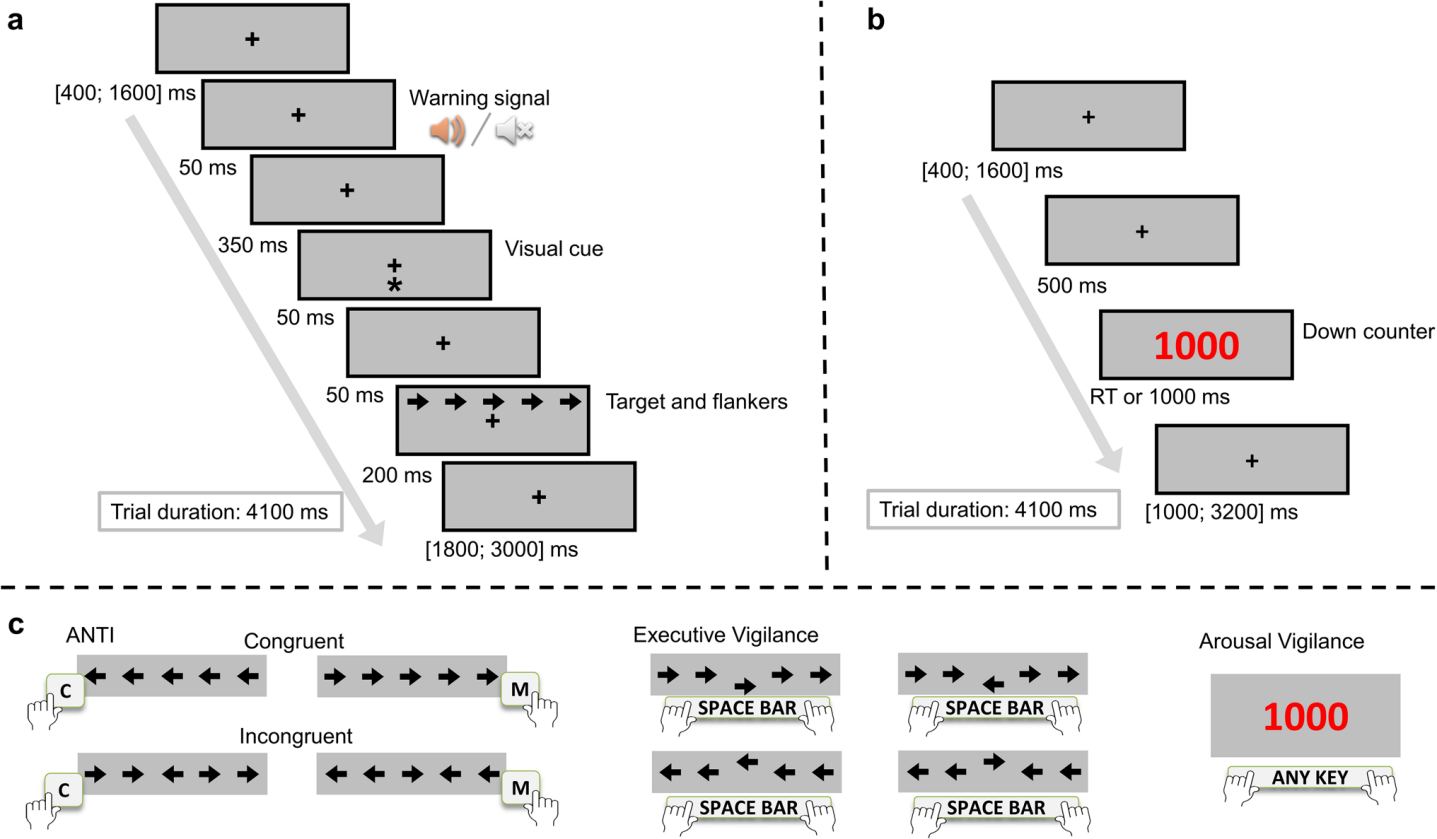
Procedure of the ANTI-Vea task. **a** Stimuli sequence and timing for the ANTI and EV trials. **b** Stimuli sequence and timing for the AV trials. **c** Correct responses expected for the ANTI (see examples of congruency condition), EV, and AV trials. In all the trials, the first and the last screen have a random timing to set the total trial duration in 4,100 ms. All responses are allowed until 2,000 ms from the target appearance

The ANTI-Vea comprises three subtasks, each of them performed in a specific set of trials that are randomly presented: ANTI (60%, a flanker paradigm combined with an auditory warning signal and a validity cueing paradigm that is suitable to assess the classic attentional functions), EV (20%, a signal-detection task similar to the SART suitable to assess the EV decrement), and AV (20%, a vigilance task similar to the PVT suitable to assess the AV decrement). A full description of the stimuli procedure and timing of each type of trial of the ANTI-Vea can be reviewed in detail in previous studies (Luna, Barttfeld, et al., 2021a; Luna et al., 2018; Luna, Roca, et al., 2021b) and in Fig. 1.

In short, the ANTI trials follow the procedure of the ANTI task (Callejas et al., 2004). Participants must respond to the direction (left/right) pointed by the target (i.e., the central arrow) while ignoring the direction pointed by the surrounding flanking arrows (see Fig. 1c). Note that, as depicted in Fig. 1a, the target and flankers can be anticipated by a warning signal (suitable to assess phasic alertness) and by a visual cue (suitable to assess attentional orienting). The flanker subtask embedded in these ANTI trials was used in the present study for measuring executive control. Thus, to assess executive control, in half of the ANTI trials the target points in the same direction as the flanking arrows (congruent condition), whereas in the other half it points in the opposite direction (incongruent condition). Cognitive control was measured as the interference effect, computed as the difference in performance between incongruent and congruent conditions.

EV trials follow the same procedure as the ANTI ones (see Fig. 1a). Participants are instructed to stay vigilant at all times to detect, by pressing a different response key, a large vertical displacement of the target (i.e., 8 px, either upwards or downwards) from its central position (see Fig. 1c), while ignoring the direction the target pointed to. Importantly, the EV trials were used for measuring the EV component, as these trials represent the embedded signal-detection subtask. Thus, in the EV trials, participants might hit or miss the occurrence of the infrequent critical signal (i.e., the vertically displaced target). Lastly, in the AV trials, no warning signal nor visual cue (nor arrows stimuli) is presented after the initial fixation point (see Fig. 1b). Instead, a red millisecond down counter starting from 1,000 appears in the AV trials and participants are instructed to stop it as fast as possible by pressing any available key from keyboard (see Fig. 1c). Therefore, the AV trials are suitable for measuring the AV component, as these trials represent the embedded RT subtask. RT scores (i.e., mean and variability of RT, and lapses as RT ≥ 600 ms) were computed as measures of the AV component (Basner & Dinges, 2011).

The standard ANTI-Vea was completed in the typical lab conditions (i.e., into an experimental room and using headphones to hear the warning signal stimuli). Participants who performed the online ANTI-Vea completed the task on a computer device with internet connection in a place of their own choosing outside the lab. Importantly, before the instructions to perform the online ANTI-Vea, participants received some additional instructions to avoid any possible distractions that are usually controlled in the lab (e.g., to turn off any entertaining device such as radio or television and to set their mobile phone on mute mode without the vibration function). In addition, they were asked to set the volume of their computer at 75% and were encouraged that if they had to move from their seat for any reason, to try to do it before starting the task. All participants received the standard instructions of the task gradually and completed several practice blocks to correctly perform each type of trial. The practice section of the task comprised three blocks with visual feedback and one block (with half of trials of an experimental block) without visual feedback (see further details on instructions and practice in Luna et al., 2018).

After practice, participants completed six experimental blocks with no pause nor visual feedback. Each block comprised 80 randomized trials (48 ANTI, 16 EV, and 16 AV). The 48 ANTI trials had the following factorial design: Warning signal (no tone/tone) × Visual cue (invalid/no cue/valid) × Congruency (congruent/incongruent) × Target direction (left/right) × Arrows position regarding the fixation point (down/up). In the EV trials, one factor was added to the factorial design: displacement direction (upwards/downwards). The 16 EV trials on each experimental block were randomly selected from the 96 possible levels of the factorial design. Each experimental block lasted 5:28 min, for a total duration of 32:48 min.

### Statistical analyses

Following standard criteria (Luna, Barttfeld, et al., 2021a; Luna, Roca, et al., 2021b), 28 participants (4.54%) were excluded from data analyses: 26 participants due to an extreme overall mean RT or percentage of errors in the ANTI trials (i.e., 2.5 *SD* above or below the sample mean) and two participants due to technical issues during data acquisition. Thus, the final sample included 589 participants. Using G*Power 3.1.9.7 (Faul et al., 2007), sensitivity analysis showed that considering a significance level of α = .05 and a power of 1 − β = .95, the minimum effect size that could be detected with the current sample (*N* = 589) for the executive control decrement across blocks (measured by the Block × Congruency interaction) was equal to *f* = .06 (which corresponds to a ${\eta}_p^2$ of .004). RStudio (R Core Team, 2021; RStudio Team, 2020) was used to conduct analysis of variance with the afex package (Singmann et al., 2021) and split-half reliability analyses (see Supplementary Material) with plyr (Wickham, 2011) and Hmisc (Harrell, 2021) packages. Correlational analyses (see Supplementary Material) were conducted with JASP (JASP Team, 2019). Figures were created using Matplotlib (Hunter, 2007).

#### Performance across time-on-task

The executive control decrement was analyzed by mean correct RT, percentage of errors, and the IE score computed from ANTI trials as a function of congruency and block conditions. For mean correct RT, following Luna, Barttfeld, et al. (2021a), trials with RT below 200 ms and above 1,500 ms (1.68%) and those with incorrect response (4.84%) were excluded from data analyses. Note that the IE score combines RT and accuracy to provide an appropriate summary of performance (Bruyer & Brysbaert, 2011; Vandierendonck, 2017). The main advantage of this score is that it provides a behavioral index free of speed–accuracy trade-offs. It is calculated for each condition as the mean correct RT divided by the proportion of correct responses (IE = Mean RT/proportion of correct responses). Thus, a score in milliseconds is obtained, which is equal to the mean RT in case of perfect accuracy (i.e., a rate of 1, or 100% correct responses). For accuracy below 1 then the IE score will increase proportionally to the decrease in accuracy. Importantly, given that the IE score is very sensitive to a low accuracy (i.e., it provides high scores reflecting low performance in these cases; Vandierendonck, 2018), and because responses with accuracy below .5 would be meaningless for this condition, IE was computed only considering those experimental blocks wherein accuracy was higher than 50% (i.e., .5 rate) in the ANTI trials (thus excluding data from 0.51% of blocks).

First, the executive control decrement across time-on-task was analyzed by three separated repeated-measures analyses of variance (ANOVAs), with mean correct RT, percentage of errors, or the IE score as dependent variables, and block (six levels) and congruency (congruent/incongruent) as within-participant factors (see Supplementary Material), with data being collapsed across the other variables.^1^ Here, aiming at providing stronger and specific evidence regarding the executive control decrement across time-on-task, three separated repeated-measures ANOVAs were conducted on interference (i.e., incongruent minus congruent conditions) measures, with mean correct RT, percentage of errors, or the IE score, as dependent variable, and blocks (6 levels) as a within-participant factor.

To test whether executive control and vigilance decrements are associated, we computed split-half reliability scores for the linear slope of the IE score as well as for vigilance dependent variables and then performed Pearson and Bayesian correlations among executive control and vigilance slopes. Noting that, as already mentioned, the observed Pearson correlations were relatively small, and also that reliability of the slope of the IE score was low, we decided to reanalyze data through a different data analysis approach to further test the predictions of interest in the present study about resource-control theory. Thus, correlational analyses and split-half reliabilities are reported in Supplementary Material, and the role of executive control on the vigilance decrement was analyzed as described below.

To specifically analyze whether the vigilance decrement (already observed in Luna, Roca, et al., 2021b) was modulated by the change in executive control across time-on-task, repeated-measures analyses of covariance (ANCOVAs) were conducted. In all ANCOVAs, executive control change (i.e., the slope of the linear change in IE interference across blocks) was included as a covariate. The slope of the linear change was computed for each participant by performing a linear model with IE interference score as dependent variable and blocks as terms of the model. These data analyses were necessary to examine whether EV/AV decrements are modulated by the change of executive control across time-on-task. ANCOVAs included either hits in EV trials, or mean RT, *SD* of RT, or the percentage of lapses (i.e., responses equal or higher than 600 ms, following the criteria of Luna, Barttfeld, et al., 2021a) in the AV trials, as dependent variable, and blocks (six levels) as a within-participant factor.

Finally, to specifically examine the modulation of executive control decrement on the vigilance decrement, mixed ANOVAs were conducted. To this end, the sample was divided into tertiles according to the size of the linear slope in the IE score (i.e., the size of the executive control change across blocks). Only the two extreme groups were included as levels of the between-participant factor in mixed ANOVAs, in particular: one group with those participants with a large decrement in executive control (i.e., all with a positive slope), and the other group with those participants in which executive control showed no decrement across blocks (i.e., all with a negative slope; please see the distribution and tertiles division of the sample according to the size of the IE slope in Supplementary Fig. 1). Note that mixed ANOVAs were necessary to disentangle the direction of the significant interactions between executive control change and EV/AV decrements observed in repeated-measures ANCOVAs. Mixed ANOVAs were modeled with those dependent variables in which a significant interaction was observed in repeated-measures ANCOVAs (i.e., hits for EV, and mean RT or *SD* of RT for AV), including blocks as within-participant factor.

All analysis of variance are reported with ${\eta}_p^2$ as a measure of the effect size and 95% confidence intervals around them (Cumming, 2014; Kelley & Preacher, 2012). Statistical significance was established at *p* < .05. As in all analysis of variance the sphericity assumption was violated, demonstrated by the significance of Mauchly’s test (all *p*s < .05), Greenhouse–Geisser correction was applied to report the statistics and degrees of freedom. Finally, planned polynomial contrasts were conducted with the emmeans package (Lenth, 2021) in RStudio to analyze the significance of the linear component across blocks in all dependent variables. Note that ${\eta}_p^2$ for planned contrasts is reported with one-sided 95% confidence intervals (i.e., with the lower estimated interval and the maximum interval equal to 1), as computed with the effectsize package (Ben-Shachar et al., 2020) in RStudio.

## Results

### Executive control decrement across time-on-task

**Fig. 2:**
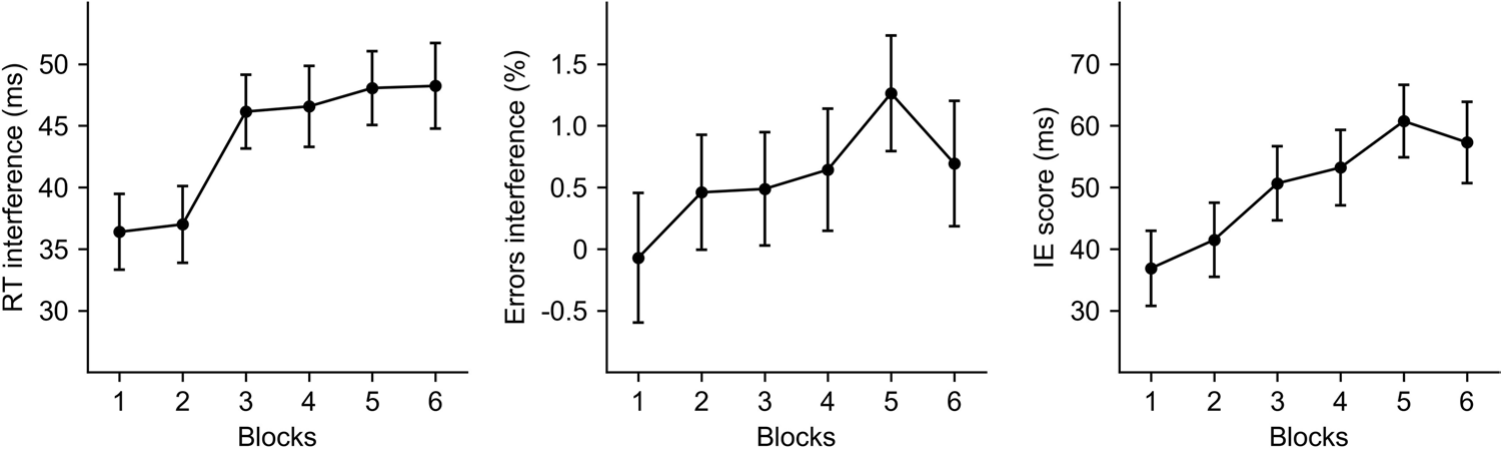
Executive control decrement across time-on-task. The interference effect across blocks is illustrated as mean reaction time (RT; left graph), percentage of errors (center graph), and the inverse efficiency (IE) score (right graph). Error bars represent 95% confidence intervals of the mean and were computed following the method developed by Cousineau (2005)

Executive control decrement was observed as a significant increase in the interference effect for mean RT, *F*(4.86, 2852.72) = 9.69, *p* < .001, ${\eta}_p^2$ = .02, 95% CI [.01, .02], percentage of errors, *F*(4.91, 2885.48) = 2.50, *p* = .029, ${\eta}_p^2$ < .01, [.00, .01], and the IE score, *F*(4.84, 2789.60) = 8.33, *p* < .001, ${\eta}_p^2$ = .01, [.00, .02], across blocks (see Fig. 2). Polynomial contrasts demonstrated that the linear increase of the interference effect across blocks was significant for mean RT, *t*(587) = 5.86, *p* < .001, ${\eta}_p^2$ = .06, 95% CI [.03, 1.00], percentage of errors, *t*(588) = 2.63, *p* = .009, ${\eta}_p^2$ = .01, [.00, 1.00], and the IE score, *t*(576) = 5.42, *p* < .001, ${\eta}_p^2$ = .05, [.02, 1.00], thus demonstrating a progressive drop in executive control across time-on-task.

### Modulation of change in executive control over the vigilance decrement

**Fig. 3:**
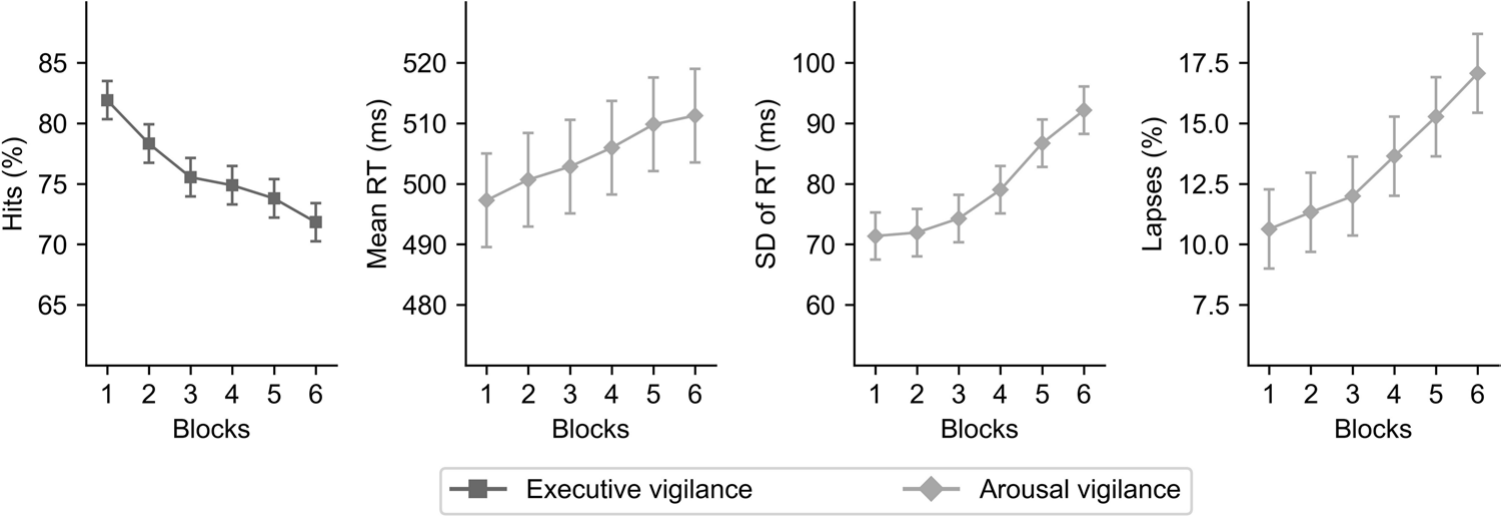
Executive and arousal vigilance decrement across time-on-task, including as covariate the linear slope across blocks of the inverse efficiency score for executive control. For EV, the decrease in hits across blocks is depicted in the left graph. For AV, the increment in mean RT (center-left graph), standard deviation (*SD*) of RT (center-right graph), and percentage of lapses (right graph) across blocks are represented. Error bars represent 95% confidence intervals

For the EV component, as depicted in Fig. 3, repeated-measures ANCOVAs showed a significant drop in hits across blocks, *F*(4.57, 2677.14) = 44.41, *p* < .001, ${\eta}_p^2$ = .07, [.05, .09]. Polynomial contrasts demonstrated a significant linear component in hits across blocks, *t*(586) = −12.66, *p* < .001, ${\eta}_p^2$ = .21, [.17, 1.00)]. Importantly, the decrement in hits showed a significant—albeit small—modulation by the executive control decrement, *F*(4.57, 2677.14) = 4.21, *p* = .001, ${\eta}_p^2$ = .01, [.00, .01].

For the AV component, as observed in the same Fig. 3, repeated-measures ANCOVAs showed a significant increase in mean RT, *F*(3.52, 2063.77) = 16.05, *p* < .001, ${\eta}_p^2$ = .03, [.02, .04], *SD* of RT, *F*(4.25, 2492.41) = 22.79, *p* < .001, ${\eta}_p^2$ = .04, [.02, .05], and the percentage of lapses, *F*(3.66, 2143.33) = 33.93, *p* < .001, ${\eta}_p^2$ = .05, [.04, .07], across blocks. Polynomial contrasts demonstrated that the linear increase was significant for all dependent variables: mean RT, *t*(586) = 5.93, *p* < .001, ${\eta}_p^2$ = .06, [.03, 1.00], *SD* of RT, *t*(586) = 8.82, *p* < .001, ${\eta}_p^2$ = .12, [.08, 1.00], and the percentage of lapses, *t*(586) = 9.62, *p* < .001, ${\eta}_p^2$ = .14, [.10, 1.00]. Importantly, executive control decrement significantly modulated—although with a relative small effect size—the AV decrement in mean RT, *F*(3.52, 2063.77) = 4.02, *p* = .005, ${\eta}_p^2$ = .01, [.00, .01], and *SD* of RT, *F*(4.25, 2492.41) = 3.32, *p* = .009, ${\eta}_p^2$ = .01, [.00, .01], but not in the percentage of lapses, *F*(3.66, 2143.33) = 1.75, *p* = .142, ${\eta}_p^2$ < .01, [.00, .01].

**Fig. 4:**
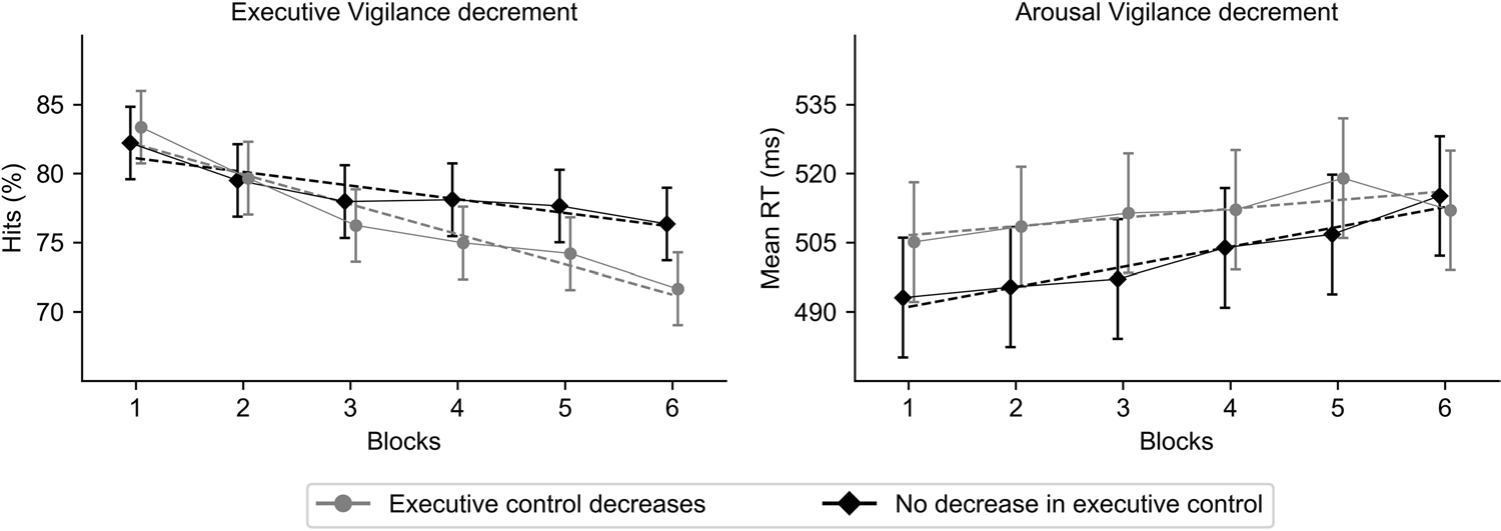
Executive (left graph) and arousal (right graph) vigilance decrement as a function of executive control performance across time-on-task. The dotted lines represent the linear trend for that group in each graph. Error bars represent 95% CI around the mean

To characterize the modulation of executive control performance across time-on-task on vigilance components, mixed ANOVAs including the two extreme groups were conducted. Importantly, the decrement in hits was significantly different between these groups that varied in their executive control performance across time-on-task, *F*(4.52, 1764.37) = 3.59, *p* = .004, ${\eta}_p^2$ = .01, [.00, .02]. As depicted in Fig. 4 (left panel), planned comparisons of the linear component showed a significant difference between the two extreme sample groups *t*(390) = 3.31, *p* = .001, ${\eta}_p^2$ = .03, [.01, 1.00]: the linear decrement in hits was larger for the group in which executive control decreases *t*(390) = −8.63, *p* < .001, ${\eta}_p^2$ = .16, [.11, 1.00] than for the group in which executive control did not decrease *t*(390) = −3.94, *p* < .001, ${\eta}_p^2$ = .04, [.01, 1.00], across blocks.

In the AV component, the decrement between groups who differed in their executive control performance across time-on-task was significantly different for mean RT, *F*(3.41, 1329.10) = 3.10, *p* = .021, ${\eta}_p^2$ = .01, [.00, .02], but not for *SD* of RT, *F*(4.11, 1603.51) = 1.02, *p* = .399, ${\eta}_p^2$ < .01, [.00, .01]. As observed in the same Fig. 4 (right panel), planned comparisons of the linear component for mean RT showed a non-significant difference between the two extreme sample groups *t*(390) = 1.86, *p* = .063, ${\eta}_p^2$ = .01, [.00, 1.00]. Furthermore, the observed non-significant effect was, however, contrary to the predictions by the resource-control theory, with the linear increase in mean RT being smaller for the group in which executive control decreased *t*(390) = 2.08, *p* = .038, ${\eta}_p^2$ = .01, [.00, 1.00], than for the group in which executive control did not decrease, *t*(390) = 4.72, *p* < .001, ${\eta}_p^2$ = .05, [.02, 1.00], across blocks.

## Discussion

The present study aimed at testing some of the critical predictions of the resource-control theory (Thomson et al., 2015) about the vigilance decrement phenomenon (Davies & Parasuraman, 1982; Helton & Warm, 2008; Mackworth, 1948, 1950). According to this theory, an executive control decline should be observed along with the vigilance decrement across time-on-task. To this end, data from a large number of participants (*N* = 617) from Luna, Roca, et al. (2021b) who performed the ANTI-Vea (Luna et al., 2018) were reanalyzed. Note that, importantly, the ANTI-Vea is a suitable task to assess the classic attentional networks functioning—which includes executive control (Petersen & Posner, 2012; Posner & Petersen, 1990)—while simultaneously measuring the EV and AV decrement (Luna et al., 2018).

Differently from Zholdassova et al. (2021) who assessed executive control with the ANT (which is not suitable to measure vigilance decrement), we observed a linear decline in executive control across time-on-task in RT, errors, and the IE score. Particularly important for the present study is that the linear decline in hits for the EV component of vigilance (Luna, Roca, et al., 2021b) was significantly modulated by the executive control decrease across time-on-task. Importantly, the EV decrement was steeper in participants wherein executive control decreased than in those participants in which executive control did not decrease across time-on-task. This set of outcomes, although correlational, fits well with the resource-control theory, which predicts that reductions in executive control impair vigilance performance across time-on-task. To the best of our knowledge, although previous research has indirectly examined the resource-control theory (Satterfield et al., 2019; Zholdassova et al., 2021), this is the first empirical study that presents critical evidence supporting the correspondence of a decrease in (executive) vigilance along with a decrease in executive control across time-on-task.

However, the outcomes related to the AV component of vigilance seem to be relatively inconsistent (see also Supplementary Material). Executive control performance across time-on-task only modulated AV for mean RT (and, as can be seen in Fig. 4, this modulation seems to be specific of the last block), but not for RT variability or lapses. Furthermore, we observed that the group in which executive control did not decrease across time-on-task is the one with the largest AV decrement, contrary to the predictions of the resource-control theory.

In an attempt to clarify this contradictory pattern of outcomes across the two vigilance measures, datasets gathered in a separated study (*N* = 340; Cásedas et al., 2022) conducted with the online ANTI-Vea to investigate the relationship between attention and vigilance with mindfulness trait (available at https://osf.io/374rs/), were reanalyzed in the same way as in the present study. The modulation of EV by executive control performance across time-on-task was replicated, *F*(4.59, 986.50) = 2.49, *p* = .034, ${\eta}_p^2$ = .01, [.00, .02]: the group with executive control decrement showed a larger linear EV decrement than the group with no-decrement in executive control, *t*(215) = 2.53, *p* = .012, ${\eta}_p^2$ = .03, [.00, 1.00]. However, for the AV component, data from this independent study showed that executive control modulated AV for variability of RT, *F*(3.61, 777.05) = 3.49, *p* = .010, ${\eta}_p^2$ = .02, [.00, .03], and for lapses, *F*(3.15, 677.60) = 3.13, *p* = .023, ${\eta}_p^2$ = .01, [.00, .03], but not for mean RT (*F* < 1), contrary to the outcomes of the present study. In this case the direction of the modulation was congruent with the hypothesis of the resource-control theory (i.e., a larger AV decrement in the group with larger executive control decrement), both for variability of RT, *t*(215) = −2.29, *p* = .023, ${\eta}_p^2$ = .02, [.00, 1.00], and lapses, *t*(215) = −2.53, *p* < .001, ${\eta}_p^2$ = .03, [.00, 1.00].

Altogether, the outcomes observed in the present study and in the one conducted by Cásedas et al. (2022) seem to provide consistent evidence regarding the modulation of the EV decrement by the executive control decrement but rather inconsistent evidence concerning the AV decrement. Indeed, this evidence aligns with the correlational analyses presented in Supplementary Material. We observed a significant negative—albeit small—Pearson correlation between the linear slopes of IE score for executive control and hits in EV, which was also supported by Bayesian correlational analyses as strong evidence in favor of the existence of a correlation. In contrast, we observed relative independence of the executive control decrement with the AV decrement in mean RT—for which Bayesian evidence indeed supported an absence of correlation—but not with the decrement in RT variability—wherein at least substantial evidence in favor of a correlation was observed.

It should be noted that, although the present study analyzed data from a large sample size, we reckon that most of the reliability indices for slopes reported in Supplementary Material were observed as problematic—particularly the IE score one—which might explain the relatively small size of the observed Pearson’s correlations among linear slopes. To further address the modulation of executive control decrement over the vigilance decrement, as hypothesized by resource-control theory, ANCOVAs and mixed ANOVAs were performed. It must be noted that these analyses are neither exempt from limitations. Both ANCOVAs and mixed ANOVAs might also be affected by the low reliability of the IE slope score. This might be particularly problematic for difference scores computed across blocks, as the reliability of overall scores in the ANTI-Vea seems to be higher than those of differential measures (Coll-Martín et al., 2021; Luna, Roca, et al., 2021b). Furthermore, mixed ANOVAs excluded one third of the sample and interindividual variability was reduced by categorizing participants in groups. We tried to address some of these limitations by performing both ANCOVAs and mixed ANOVAs, which have unique limitations. On the one hand, the limitations associated with categorizing participants were not applicable to the ANCOVAs, which included all participants in the analysis. On the other hand, although sample size was reduced in mixed ANOVAs by analyzing only the two extreme groups, it should be noted that the sample size was still large in each group (i.e., ~ 196 participants per group).

Regarding the inconsistency of outcomes observed for EV and AV, this might be accounted by the different mechanisms underlying vigilance components. Whereas AV might be a component more related to the arousal mechanisms of attention, EV might be rather considered as a goal-directed component of vigilance. In this vein, prior research has shown that AV is particularly related to the phasic alertness state (Luna, Roca, et al., 2021b) and modulated by the changes in the sleep–wake cycle (Lim & Dinges, 2008) and stimulants such as caffeine (Sanchis et al., 2020), whereas EV scores (i.e., hits, false alarms, and sensitivity and response bias indices) were specifically correlated with the interference score of executive control (Luna, Roca, et al., 2021b) and the decrement in hits was mitigated by transcranial stimulation of the right frontoparietal attentional network (Luna, Román-Caballero, et al., 2020b). In line with these findings, the present outcomes seem to support that EV is relatively more associated with the executive control decrement than AV, but future studies should more deeply examine this critical issue.

It is worth noting that, although the modulation observed by executive control performance across time-on-task over the EV decrement seems to fit well with the resource-control theory, the effect sizes observed in the present experiment are rather small. In the same vein, the correlation between slopes of hits for EV and the IE score for executive control reported in Supplementary Material, albeit statistically significant, is also small (*r* = −.14). Furthermore, note that even participants in the group who did not show any decrement in executive control across time-on-task (some of them showed in fact a progressive increment) still showed a significant decrement in EV. All this evidence points to the fact that the outcomes observed from the present data, which—importantly—were gathered from a large sample size, seem to explain only a small part of the full variance. This is an important aspect of the present results since the resource-control theory highlights the importance of the decrease in executive control as the main reason for the vigilance decrement. Thus, such small effect sizes are controversial when it comes to concluding evidence in favor of this theory as it is currently framed. Conversely, the present results raise the need to consider other unknown factors apart from the decrease in executive control as contributing to the vigilance decrement. One of the potential variables to consider could be motivation (Hockey, 1997). In this vein, Reteig et al. (2019) showed that after 60 minutes of performing a task, the vigilance decrement was partially reduced after a monetary unexpected reward, and Esterman et al. (2016) alleviated the vigilance decrement by anticipating a large monetary loss given an error, increasing the cost of error and the benefit of sustaining attention (Kurzban et al., 2013). Introducing a motivational manipulation during the performance of ANTI-Vea could shed light on this question.

Furthermore, it should be noted that no causal inference can be derived from the present results. Given that the resource-control theory explains the vigilance decrement as a consequence of the executive control depletion, future studies should test this theory in a causal manner, for instance, using noninvasive stimulation techniques such as transcranial direct current stimulation or transcranial magnetic stimulation (Rossini et al., 2015; Yavari et al., 2018) to hinder executive control and assess whether this leads to larger vigilance decrements, as done in other studies to assess the role of cognitive control in implicit learning (Prutean et al., 2021). Besides, note that the resource-control theory predicts that resources are misappropriated by mind-wandering (Smallwood & Schooler, 2006). Because we reanalyzed already gathered data, no mind-wandering measures were available. Importantly, we consider the current research as a first step into testing some of the predictions of the resource-control theory, by measuring—to the best of our knowledge—for the first time both the vigilance and the executive control decrement simultaneously. Thus, future studies should consider adding mind-wandering measures to the ANTI-Vea task to further examine the predictions derived from the resource-control theory, for instance, by introducing thought probes within the task (Seli et al., 2018; Thomson et al., 2014) or by measuring changes in physiological indices (Arnau et al., 2020; Konishi et al., 2017) as proxy measures of mind-wandering. Finally, one major challenge for future studies would be to improve reliability in the score used to assess executive control change across time-on-task—which would solve some of the limitations observed in the present study—while still simultaneously measuring the vigilance decrement within the same task.

To conclude, the present study presents novel evidence regarding some of the predictions stated by the resource-control theory. In particular, using a suitable task for simultaneously measuring the vigilance decrement phenomenon and changes in executive control across blocks, we provide evidence for the first time that executive control decreases across time-on-task along with EV. Importantly, the EV decrement was larger in those participants wherein executive control decreases than in those participants in which executive control does not decrease across time-on-task. This set of outcomes provides partial support for the resource-control predictions about the vigilance decrement phenomenon. However, given the small effect sizes observed in datasets gathered from a large sample size, together with the fact that the relationship was consistently observed only for EV but not for AV, we can conclude that there must be additional variables, not considered by the resource-control theory, explaining the vigilance decrement. Future research should also study causal mechanisms of the executive control decrement on the changes of EV and AV across time-on-task and the role of mind-wandering on the resource-control predictions.

## Supplementary Information

ESM 1 (DOCX 72 kb)

## Data Availability

The datasets analyzed during the current study are available in the Open Science Framework repository (http://osf.io/q85bu). The standard ANTI-Vea is publicly available in the Open Science Framework repository (https://osf.io/4n2kx/), and the online ANTI-Vea is publicly available online (https://www.ugr.es/~neurocog/ANTI/).

## Footnotes

1 The statistical analysis of the main effects and interactions of warning signal, visual cue, and congruency factors, and the modulations by the task version, can be reviewed in detail in *`*Luna et al. (2021b).

## Code availability

The script code for generating processed data tables and performing statistical analyses is available in the Open Science Framework repository (https://osf.io/2vmt7/).
