# Supplementary material for: A vigilance decrement comes along with an executive control decrement: Testing the resource-control theory
Source: Psychon Bull Rev. 2022 Apr 27;29(5):1831–43. doi: 10.3758/s13423-022-02089-x (PMC9568446; doi:10.3758/s13423-022-02089-x)
Supplement: Supplementary file 1 — (DOCX 72 kb) [file 13423_2022_2089_MOESM1_ESM.docx]

## Supplementary Material

### Executive control decrement as a function of congruency conditions and blocks

The main effect of congruency was significant for reaction time (RT) [*F* (1, 587) = 1100.51, *p* < .001, $\eta_{p}^{2}$ = .65, 95% CI (.61, .69)] and errors [*F* (1, 588) = 10.37, *p* = .001, $\eta_{p}^{2}$ = .02, (.00, .04)]. Responses were faster and more accurate in the congruent (RT: *M =* 632 ms, 95% CI [623, 640]; errors *M =* 5.75%, [5.35, 6.16]) than incongruent (RT: *M =* 675 ms, [667, 683]; errors *M =* 6.33%, [5.93, 6.74]) condition. The main effect of blocks was significant for RT [*F* (3.57, 2094.56) = 13.91, *p* < .001, $\eta_{p}^{2}$ = .02, (.01, .04)] although not for errors [*F* (3.89, 2289.71) = 1.55, *p* = .186, $\eta_{p}^{2}$ < .01, (.00, .01)] (see Supplementary Table 1). However, as detailed in Supplementary Table 1, congruency significantly varied across blocks both for RT [*F* (4.86, 2852.72) = 9.69, *p* < .001, $\eta_{p}^{2}$ = .02, (.00, .03)] and errors [*F* (4.91, 2885.48) = 2.50, *p* = .030, $\eta_{p}^{2}$ < .01, (.00, .01)]. The same pattern of results was observed for the inverse efficiency (IE) score, as shown in Supplementary Table 1: responses were more efficient for congruent (*M =* 674, [664, 684]) than incongruent (*M =* 723, [713, 733]) trials, [*F* (1, 576) = 442.35, *p* < .001, $\eta_{p}^{2}$ = .43, (.38, .48)], and there was a significant main effect of blocks, [*F* (3.78, 2178.87) = 8.15, *p* < .001, $\eta_{p}^{2}$ = .01, (.00, .02)]. Importantly, as with RT and errors, congruency also increased across blocks for the IE score, [*F* (4.84, 2789.60) = 8.33, *p* < .001, $\eta_{p}^{2}$ = .01, (.00, .02)].

**Supplementary Table 1.** Mean correct RT, percentage of errors, and IE score across blocks as a function of congruency conditions.

|  |  | Congruent | |  | Incongruent | |
| --- | --- | --- | --- | --- | --- | --- |
|  |  | *M* | 95% CI |  | *M* | 95% CI |
| Reaction Time (ms) | |  |  |  |  |  |
|  | Block 1 | 648 | [639, 656] |  | 684 | [675, 693] |
|  | Block 2 | 637 | [628, 645] |  | 674 | [665, 682] |
|  | Block 3 | 629 | [620, 638] |  | 675 | [666, 684] |
|  | Block 4 | 626 | [617, 635] |  | 672 | [663, 681] |
|  | Block 5 | 624 | [615, 633] |  | 672 | [663, 681] |
|  | Block 6 | 626 | [617, 635] |  | 675 | [666, 683] |
| Errors (%) | |  |  |  |  |  |
|  | Block 1 | 6.15 | [5.56, 6.75] |  | 6.08 | [5.49, 6.68] |
|  | Block 2 | 5.74 | [5.14, 6.33] |  | 6.20 | [5.60, 6.79] |
|  | Block 3 | 5.62 | [5.03, 6.22] |  | 6.11 | [5.52, 6.71] |
|  | Block 4 | 5.42 | [4.82, 6.01] |  | 6.06 | [5.47, 6.66] |
|  | Block 5 | 5.45 | [4.86, 6.05] |  | 6.72 | [6.12, 7.31] |
|  | Block 6 | 6.13 | [5.53, 6.72] |  | 6.82 | [6.22, 7.41] |
| Inverse Efficiency | |  |  |  |  |  |
|  | Block 1 | 695 | [683, 706] |  | 730 | [719, 742] |
|  | Block 2 | 679 | [668, 691] |  | 720 | [709, 732] |
|  | Block 3 | 671 | [659, 682] |  | 721 | [709, 732] |
|  | Block 4 | 665 | [653, 677] |  | 717 | [705, 728] |
|  | Block 5 | 664 | [652, 676] |  | 723 | [711, 735] |
|  | Block 6 | 669 | [658, 681] |  | 726 | [714, 737] |

*Note*. *M* = mean, CI = confidence intervals, ms = milliseconds.

### Split-half reliability scores of executive control and vigilance slopes across time-on-task

Reliability of executive control, EV, and AV decrement across time-on-task was measured by analyzing the split-half reliability of linear slopes across blocks. The slope of the linear change was computed for each participant by performing a linear model with either IE interference score for executive control, hits for EV, or mean RT, *SD* of RT, or the percentage of lapses for AV, as dependent variable, and blocks as terms of the model. Following the analysis performed by Luna, Roca, Martín-Arévalo, & Lupiáñez (2021), the split-half reliability of the linear slope was computed on the following dependent variables: (a) IE score as a summary measure of executive control, (b) hits for EV, and (c) mean RT, *SD* of RT, and percentage of lapses for AV. Note that, given that we were interested on reliability of vigilance decrement measures, differently from Luna et al. (2021), the experimental trials of each participant were randomly split on two halves at the block level. This procedure was repeated by a permutation approach until 10,000 pairs of trials’ halves were obtained. Next, for each permutation, we computed the linear slope on the IE, hits, and AV scores above-mentioned in each of the two halves. Pearson correlations across participants were performed between the two halves for each dependent variable. The split-half reliability indices were obtained as the average of the 10,000 correlations of each linear slope. Importantly, to extrapolate test-retest reliability from split-half indices, we applied the Spearman-Brown prophesy formula on Pearson correlations’ mean (MacLeod et al., 2010). The size of reliability indices can be interpreted following Draheim, Mashburn, Martin, & Engle (2019) as follows: below .70 as problematic, between .70 and .79 as borderline, and above .80 as acceptable.

Split-half reliability of each dependent measure was analyzed and are reported in Supplementary Table 2.

**Supplementary Table 2.** Descriptive statistics and mean split-half reliability correlations (Pearson’s *r*) for the executive control and EV/AV linear slopes. The Spearman-Brown formula extrapolates test-retest reliability from split-half correlations.

|  |  | Descriptive statistics |  | Split-half correlations | |
| --- | --- | --- | --- | --- | --- |
|  |  | Linear slope [95% CI] |  | Mean *r* [95% CI] | Spearman-Brown |
| Executive control | |  |  |  |  |
|  | IE score (ms) | 4.80 [3.08, 6.52] |  | .097 [.096, .098] | .177 |
| Executive vigilance | |  |  |  |  |
|  | Hits (%) | -1.85 [-2.14, -1.56] |  | .412 [.412, .413] | .583 |
| Arousal vigilance | |  |  |  |  |
|  | Mean RT (ms) | 2.86 [1.91, 3.81] |  | .621 [.620, .621] | .766 |
|  | SD of RT (ms) | 4.37 [3.39, 5.35] |  | .396 [.395, .397] | .567 |
|  | Lapses (%) | 1.30 [1.04, 1.57] |  | .585 [.584, .585] | .738 |

*Note*. *r* = Pearson correlation; CI = confidence interval; RT = reaction time; SD = standard deviation; ms = milliseconds.

### Correlations among linear slopes

To examine whether the EV and/or AV decrement is associated with a loss on executive control across time-on-task, bi-variate correlations among the linear slope of the executive control IE score, hits, for EV, and mean RT, *SD* of RT, and percentage of lapses, for AV, were conducted. First, Pearson correlations were conducted as null-hypothesis-significance-testing. The size of Pearson correlations can be interpreted following Gignac & Szodorai (2016) as follows: between .10 and .19 as small, between .20 and .29 as medium, and equal or above .30 as large correlations. Next, to test the amount of evidence gathered in favor either of the null (H_0_) or the alternative (H_1_) hypothesis, one-way Bayesian correlations were conducted. We used the standard parameters provided by JASP v.0.13.1 for Bayesian correlations, with prior including a stretched beta prior width = 1. Note that Bayesian correlations were conducted with a-priori hypothesis, i.e., to examine as H_1_ a negative correlation between the linear slopes of the executive control IE score and hits for EV, and a positive correlation between the linear slopes of the executive control IE score and mean RT, *SD* of RT, or the percentage of lapses for AV (i.e., the larger the decrement on executive control, the larger the decrement on EV/AV). The inverse Bayes Factor (BF_10_) obtained from Bayesian correlations can be interpreted following Jarosz & Wiley (2014) as follows: (a) below 0.33, as consistent evidence in favor for the H_0_; (b) between 0.33 and 1, as inconsistent evidence supporting neither the H_0_ nor the H_1_; (c) between 1 and 3, as anecdotal evidence for the H_1_; and (d) above 3, as consistent evidence supporting the H_1_, in particular: between 3 and 10 as substantial, between 10 a 30 as strong, between 30 and 100 as very strong, and higher than 100 as decisive evidence for the H_1_.

As can be observed in Supplementary Table 3, Pearson’s correlations showed as significant the expected negative correlation between the linear slopes of the executive control IE score and hits, for EV, and the expected significant positive correlations among the linear slopes of the executive control IE score and *SD* of RT and the percentage of lapses, for AV. Then, to test whether the relative small significant correlations observed in Pearson’s analysis provided at least substantial evidence in favor of the existence of a correlation, Bayesian correlations with a-priori hypotheses were conducted. Importantly, as observed in Supplementary Table 3, strong evidence in favor of the negative correlation between the linear slopes on the executive control IE score and hits, for EV, was found. Note that, as observed in Supplementary Table 3, Bayesian correlation between the linear slope on the executive control IE score and AV slopes were not similar: whereas substantial evidence in favor of a positive correlation was found for the *SD* of RT, evidence was inconsistent regarding the existence of a correlation with the percentage of lapses, and was consistent in favor of the absence of a correlation regarding mean RT. Thus, in summary, the evidence gathered provides strong evidence in favor of the association between the linear decrement on executive control and EV and at least substantial evidence in favor of the association between the linear decrement on executive control and the *SD* of RT for AV.

**Supplementary Table 3.** Bivariate Pearson and Bayesian correlations between the linear slopes of executive control and EV/AV.

| Vigilance linear slopes | | Coefficient | IE linear slope |
| --- | --- | --- | --- |
| Executive vigilance | |  |  |
|  | Hits | Pearson's *r* | -.14**** |
|  |  | BF_10_ | **44.38** |
| Arousal vigilance | |  |  |
|  | Mean RT | Pearson's *r* | -.07 |
|  |  | BF_10_ | **0.02** |
|  | SD of RT | Pearson's *r* | .12*** |
|  |  | BF_10_ | **7.48** |
|  | Lapses | Pearson's *r* | .08* |
|  |  | BF_10_ | 0.78 |

*Note*. Inverse Bayes factors (BF_10_) supporting either the H_0_ (i.e., < 0.33) or the H_1_ (i.e., > 3) are in boldface. IE = inverse efficiency score; SD = standard deviation; RT = reaction time.

* *p* < .05, ** *p* < .01, *** *p* < .005, **** *p* < .001

### Correlations between increase in interference and vigilance decrement across blocks per participant

Following Thomson, Seli, Besner, & Smilek (2014), IE interference scores across blocks were correlated with the EV/AV measures separately for each participant. The average of the correlations between the IE scores and hits was significantly negative (mean *r* = -.079, *t* (585) = -4.22, *p* < .001, *d* = -0.17, BF_10_ = 575). In contrast, for AV, the average of the correlations across blocks (per participant) between the IE scores and *SD* of RT was not significant and Bayesian evidence supported the lack of any correlation (mean *r* = .011, *t* (587) = 0.59, *p* = .723, *d* = 0.02, BF_10_ = 0.080). A similar pattern was observed for mean RT (mean *r* = -.004, *t* (587) = -0.21, *p* = .723, *d* = -0.01, BF_10_ = 0.040) and the percentage of lapses (mean *r* = .002, *t* (512) = 0.10, *p* = .539, *d* = 0.01, BF_10_ = 0.054).

### Distribution of IE slope

Supplementary Figure 1 depicts the distribution of IE slope size in the analyzed sample.


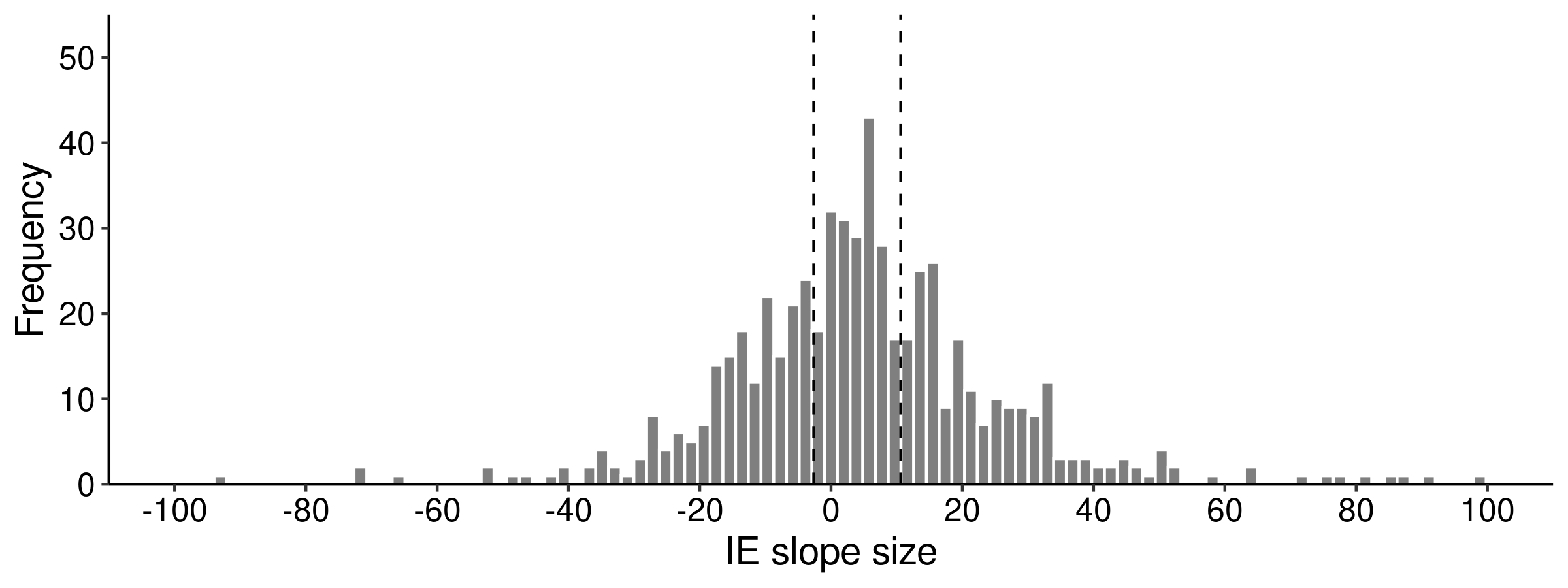
**Supplementary Figure 1.** Histogram of frequencies of the inverse efficiency (IE) slope size across the sample. Vertical dotted lines show the division of sample in tertiles, in particular: (a) from left to the first dotted line, the group with no executive control decrement; (b) between the two dotted lines, the group with relatively small executive control decrement; and (c) from the second dotted line to right, the group with large executive control decrement.

### References

Draheim, C., Mashburn, C. A., Martin, J. D., & Engle, R. W. (2019). Reaction time in differential and developmental research: A review and commentary on the problems and alternatives. *Psychological Bulletin*, *145*(5), 508–535. https://doi.org/10.1037/bul0000192

Gignac, G. E., & Szodorai, E. T. (2016). Effect size guidelines for individual differences researchers. *Personality and Individual Differences*, *102*, 74–78. https://doi.org/10.1016/j.paid.2016.06.069

Jarosz, A. F., & Wiley, J. (2014). What Are the Odds? A Practical Guide to Computing and Reporting Bayes Factors. *The Journal of Problem Solving*, *7*(1), 2–9. https://doi.org/10.7771/1932-6246.1167

Luna, F. G., Roca, J., Martín-Arévalo, E., & Lupiáñez, J. (2021). Measuring attention and vigilance in the laboratory vs. online: The split-half reliability of the ANTI-Vea. *Behavior Research Methods*, *53*(3), 1124–1147. https://doi.org/10.3758/s13428-020-01483-4

MacLeod, J. W., Lawrence, M. A., McConnell, M. M., Eskes, G. A., Klein, R. M., & Shore, D. I. (2010). Appraising the ANT: Psychometric and theoretical considerations of the Attention Network Test. *Neuropsychology*, *24*(5), 637–651. https://doi.org/10.1037/a0019803

Thomson, D. R., Seli, P., Besner, D., & Smilek, D. (2014). On the link between mind wandering and task performance over time. *Consciousness and Cognition*, *27*(1), 14–26. https://doi.org/10.1016/j.concog.2014.04.001
